# INTERNATIONAL ENVIRONMENTAL HEALTH: New MPH Program a First for India

**DOI:** 10.1289/ehp.118-a114

**Published:** 2010-03

**Authors:** Adrian Burton

**Affiliations:** **Adrian Burton** is a biologist living in Spain who also writes regularly for *The Lancet Oncology*, *The Lancet Neurology*, and *Frontiers in Ecology and the Environment*

Not often does a new degree program make headline news, but then, in India there is nothing entirely comparable to the new master’s of public health (MPH) program launched 19 January 2010 at Sri Ramachandra University (SRU) in Chennai. With environmental disease constituting roughly half the country’s total disease burden, India has a dire need of the expertise the program will develop with its focus on occupational and environmental health (OEH).

“This program is the first MPH [in India] with an exclusive focus on OEH,” says Kalpana Balakrishnan, a professor of biophysics and head of the Department of Environmental Health Engineering at SRU. “These graduates will be trained in recognition, evaluation, and management of OEH risks to serve industry and governmental organizations in addition to assuming roles as teachers of OEH in [this and other] MPH programs.”

India faces a double whammy of traditional and modern OEH problems, including indoor air pollution (primarily due to the use of wood, dung, and other solid fuels for cooking); the microbial and chemical contamination of water; chemical, physical, ergonomic, and psychosocial hazards in the workplace; and contamination of food supplies by heavy metals, pesticides, and microbes. Over 37 million people—more than the total population of California—are affected annually by waterborne disease, according to the international nonprofit WaterAid, and the most recent data available—published by James Leigh et al. in the September 1999 issue of *Epidemiology*—put the number of occupation-related deaths per year at an estimated 121,000. Yet the country, with one-sixth of humanity and a space program, nevertheless has few professionals trained to tackle such problems and inadequate capacity to undertake related research.

The 2-year MPH program is the fruit of a collaboration with the University of California, Berkeley, that began in 2002 with support from the International Training and Research Program in Environmental and Occupational Health (ITREOH), an initiative of the Fogarty International Center, National Institutes of Health, in collaboration with the NIEHS and the Centers for Disease Control and Prevention. “A main objective of this collaboration has been to build the capacity of staff at SRU to deliver the MPH program and undertake research,” explains Kirk Smith, a professor of global environmental health at Berkeley and a principal investigator with ITREOH. Fogarty funds have brought 6 SRU faculty to Berkeley to take advanced courses in biostatistics, epidemiology, and other disciplines. Berkeley aims to continue to provide training support over at least the next 2 years.

The new MPH program covers topics such as epidemiology, biostatistics, occupational toxicology, industrial hygiene, occupational and environmental safety, exposure assessment and control, health policy and management, behavioral sciences in health, indoor air quality, analysis of airborne chemical contaminants, occupational health clinics, environmental engineering (including the management of hazardous and biomedical waste), and the use of geographic information systems. With initial graduating classes of just 10 students, Balakrishnan says, “The intake will be expanded once a critical mass of teachers is produced from the first few batches.” She believes it could be at least a decade before much of a dent could be made in the subcontinent’s OEH problems and that to truly meet its challenge, India would need dozens more such programs.

“It will be difficult in the short term to find students able to pay their own fees to attend until there are jobs available to make it seem worthwhile,” Smith says. “It’s a chicken-and-egg situation, which is one of the main reasons SRU needs to start small and build up slowly to a larger program.”

Smith says other Indian universities are now considering offering new courses with OEH content, but one of the major problems they face remains the recruitment of staff capable of teaching in this area. “The production of the first ‘homegrown’ graduates who can specifically fill these positions, who have firsthand experience of India’s OEH problems, and who can begin research to find new ways of quantifying them and dealing with them will be a big step in the right direction,” he says.

“Addressing India’s occupational and environmental health concerns is vital in the future of a healthy India,” remarks Sanjay Zodpey, director of the Indian Institute of Public Health and director of public health education for the Public Health Foundation of India, both in New Delhi. “The MPH program in collaboration with Berkeley will not only address the shortfall in trained manpower in occupational and environmental health, but also strengthen indigenous Indian research capacity in these areas.”

## Figures and Tables

**Figure:**
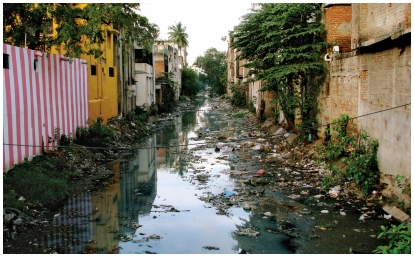
Left: Canals filled with waste and debris, like this waterway in Kanchipuram, a city known for its temples, are a common sight in India.

**Figure:**
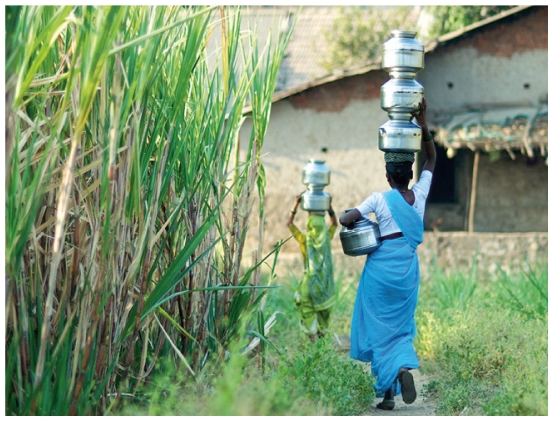
Inset: A woman from Nandgaon carries water home past the heavily fertilized sugarcane fields near her community’s open well.

